# Application of vertical transposition flap in closure for large facial soft tissue defects in children

**DOI:** 10.3389/fped.2023.1171092

**Published:** 2023-05-05

**Authors:** Rui Feng, Jigang Chen, Yining Wang

**Affiliations:** ^1^ Department of Burn and Plastic Surgery, Beijing Children’s Hospital, Capital Medical University, Beijing, China; ^2^ Department of Neonatal Surgery, Beijing Children's Hospital, Beijing, China

**Keywords:** children, facial defects, transposition flap, vertical, reconstruction

## Abstract

**Background:**

While transposition flap is widely used for the repairs of facial defects, few studies has reported its application among children with large defects. In this study, we aimed to investigate the surgical techniques and principles in different locations on face of vertical transposition flap in children.

**Methods:**

We retrospectively reviewed our hospital database and identified children who were treated with vertical transposition flap for large facial defects between January 2014 and December 2021. Information was collected including patients' demographics, location and dimension of the lesion, surgical procedure, additional surgeries, complications, and outcomes.

**Results:**

A total of 122 patients (77 boys, 63.1%) were included in this study. The average age for participants was 3.3 years (3 months to 9 years). One hundred and four (85.3%) patients had melanin nevus and 18 (14.8%) had sebaceous nevus. The average size of defects was 5.8 cm^2^ (ranging from 0.8–16.5 cm^2^). Ten patients (8.2%) suffered from dermal layer or full-thickness necrosis in the distal part of their flaps, They all recovered after conservative treatment and there were noticeable scars at discharge. Five patients (4.1%) had slight traction of the mouth and eyelid, all recovered about 2 week after surgery. An acceptable cosmetic outcome was achieved for all the patients at last time follow-up.

**Conclusions:**

Repairing large facial defects with vertical transposition flap is effective in Children, especially on forehead, cheek and mandible. However, this technique is far from perfect. Careful selection of appropriate patients and flap design might be needed.

## Introduction

Facial defects resulting from the excision of skin lesions such as melanin nevus, sebaceous nevus, or scars are relatively common in children. Face is the center of communication and emotion expression. Therefore, large lesions of the face may lead to devastating emotional and psychological outcomes among some sensitive populations such as children, who developed self-esteem and a sense of self-image at around 5 years of age (1, 2). Reconstruction of facial defects should consider cosmetic and functional results, such as skin texture, color match, and blood supply (3, 4). A local flap comprises skin and subcutaneous tissue with a direct vascular supply. It has several advantages including reliable blood supply, good skin texture and color match, and a single stage procedure (4).

Transposition flaps are among one of the most widely used local flaps for the repairs of facial defects. Larger defects can be better closed when the angle of the flap to the defect is increased to a right angle (5). Numerous studies have reported transposition flaps for reconstructing facial defects, including the area, angle, shape, and outcomes (6–9). However, few articles have discussed the surgical application in children. This article discusses the surgical techniques and application locations site of the vertical translocation flap in children.

## Methods

### Participants

A retrospective study was conducted for patients admitted to Department of Burn and Plastic Surgery, Beijing Children's Hospital, Capital Medical University, between January 2014 and December 2021 who were treated by 90° transposition flap for large facial defects. Clinical information concerning the patients' demographics, location and dimension of the lesion, surgical procedure, additional surgeries, complications, and outcomes were collected. The study was approved by the ethical review board of Beijing Children's Hospital, and informed consent was waived due to the retrospective nature of the study.

### Surgical procedure

**Figure 1:**
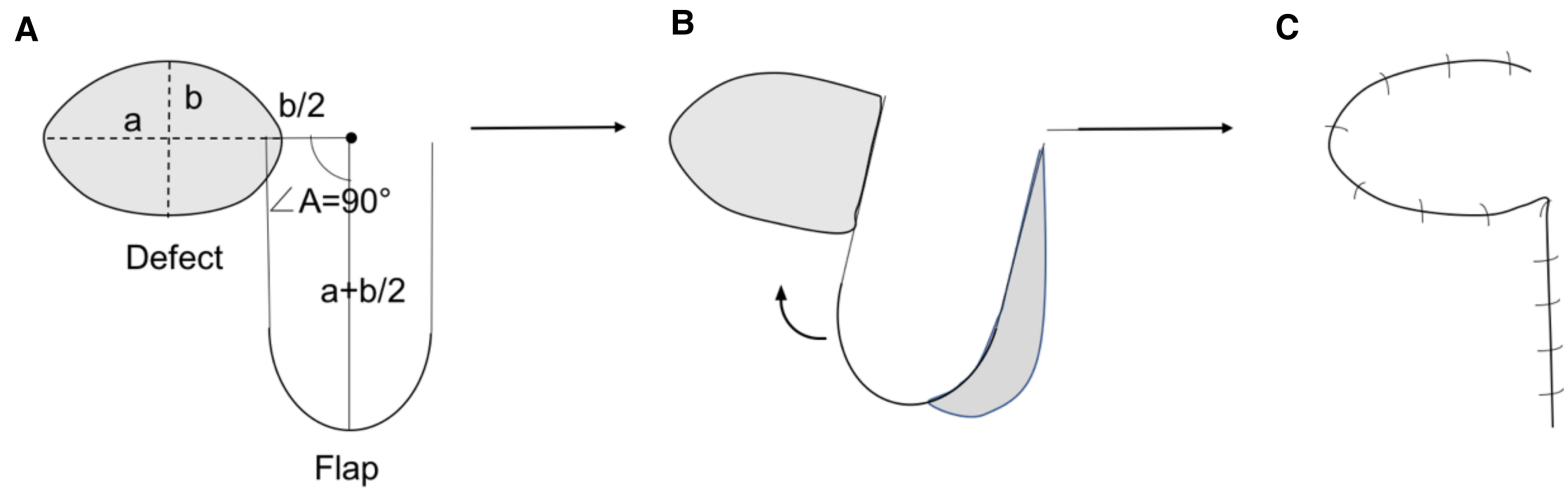
(**A**) Design of the flap. The angle A is nearly 90 degree. The length of two legs of ∠A are approximately sum of a and 2/b(a + 2/b). (**B**) Rotate the flap to cover the defect. (**C**) Close the wound from donor area.

Before surgery, the individual facial skin was examined carefully to check if the primary closure was possible. Preoperative assessment of the lesion size, laxity of surrounding tissues, the possible flap length was made for all patients. Under general anesthesia, skin lesions were excised with a margin of clinically normal appearing skin to ensure complete removal. The depth of the excision was extended into the subcutaneous tissue. After excision, the defects often exhibited a circular or oval shape. The long (a) and short (b) axis of the defect was decided (Figure 1). A line extended from the long axis was drawn and the length of this extended line was half of the short axis (b/2). Another line that was vertical to the extended line was drawn at its end and the length of this vertical line was decided at the sum of long axis and half of the short axis (a + 2/b). Then the border of the flap was designed based on the extended and vertical line, and the size of flap was slightly larger than that of defect. After that, we incised the skin along the margins of the flap and separate it at the layer of Superficial Musculo-Aponeurotic System to ensure sufficient perfusion through subcutaneous vessels. The donor site was closed first. Then the elevated flap was transferred by 90 degrees to cover the defect and sutured in different layers with tension. Minor modifications would be made during the procedure if the flap wasn't able to be transposed freely or there was a limitation in arc of rotation.

## Results

A total of 122 children were included in this study, with 77 (63.1%) being male. The average age for participants was 3.3 years (interquartile range: 3 months to 9 years). One hundred and four (85.3%) patients had melanin nevus and 18 (14.8%) had sebaceous nevus. The average size of defects was 5.8 cm^2^ (ranging from 0.8–16.5 cm^2^). Forty-eight (37.7%) patients had the defects on the cheek, 26 (27.4%) on the forehead, 22 (19.4%) on the mandible, 17 (13.9%) on the eyelid, 9 (7.4%) on the nose (Table 1).

**Table 1:** Basic information and follow-up of flaps in different locations.

	Cases	Average size (cm^2^)	Flap necrosis	Thicker flap
Cheek	48	6.6	3	3
Forehead	26	8.1	2	1
Mandible	22	5.9	1	1
Eyelid	17	2.3	0	3
Nose	9	1.3	0	0

All patients had successful lesion excision and vertical transposition flap to close the wound in single procedure. Three patients (4.8%) had incomplete eyelid closure, and 2 patients had slight traction of the upper lip immediately after surgery. All of them were fully recovered 2 week later. At discharge, one hundred and twelve (91.8%) patients had their flaps survived. Four subjects (3.2%) suffered from dermal layer necrosis with pigment differences between the flap and surrounding skins. Another six subjects (4.8%) had full-thickness skin necrosis with noticeable scars left at discharge. Eight patients (6.6%) had a thicker flap than the surrounding tissues and 2 of them underwent flap thinning one year later. The average clinical follow-up was 8 months (Range: 3 to 24 months). An acceptable cosmetic outcome was achieved for all the patients.

## Illusive case 1

**Figure 2:**
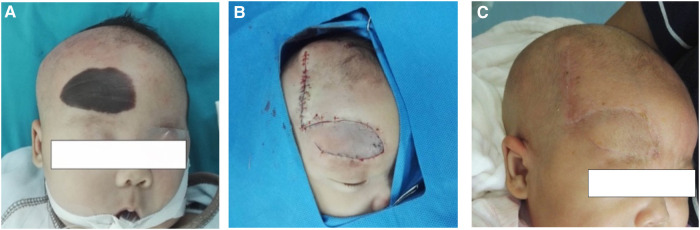
(**A**) Intraoperative lesion skin before excision. (**B**) Defect closure by 90° transposition flap. (**C**) 8 months after the surgery with light scar and pigment left because of partial-dermal necrosis of flap.

This was 3-month-old boy with melanin nevi (6.0 * 3.0 cm) on forehead. His parents requested a single surgical repair because they were concerned about the effect of multiple anesthesia on the child's central system development. He had vertical transposition flap to cover the defect. However, removal of standing cutaneous deformities (dog-ear) led to de-vascularization of the flap and the distal part of the flap suffered partial-dermal necrosis two weeks later. He recovered well at discharge after conservative treatment. Last time follow-up at 8 months after surgery showed light scar and pigment left (Figure 2). The local hairs were removed by laser therapy (Intense pulsed light).

## Illusive case 2

**Figure 3:**
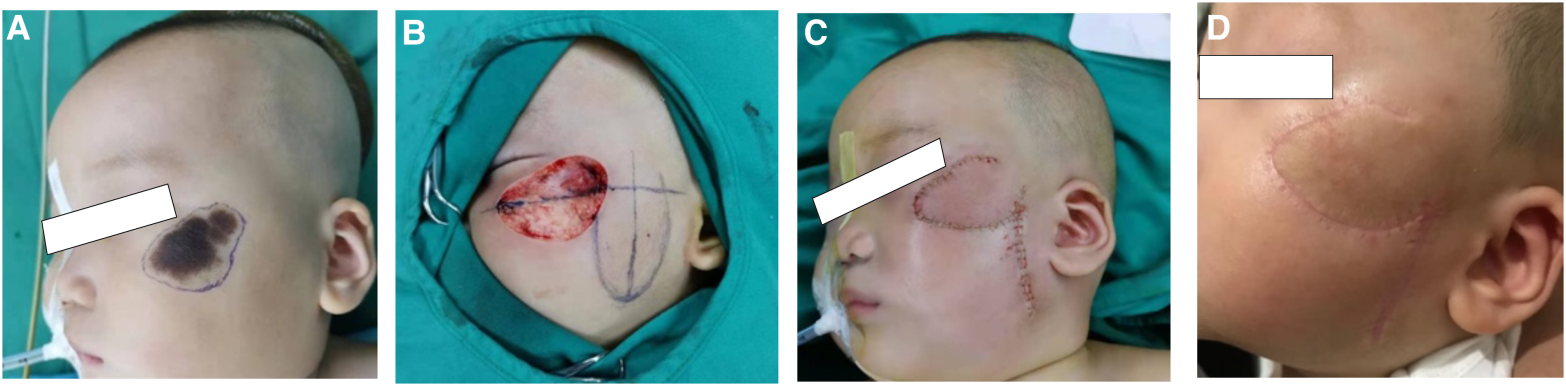
(**A**) Design of excision area preoperative. (**B,C**) Defect closure by vertical transposition flap. (**D**) 1 month after the surgery.

An 1-year-old boy presented with melanin nevi (4.0 * 3.0 cm) on the left cheek. His parents rejected serial incision, so we designed vertical transposition flap to cover the defect within the right cheek aesthetic subunit. The patient healed uneventfully, and the follow-up 1 month later showed acceptable scar (Figure 3).

## Illusive case 3

**Figure 4:**
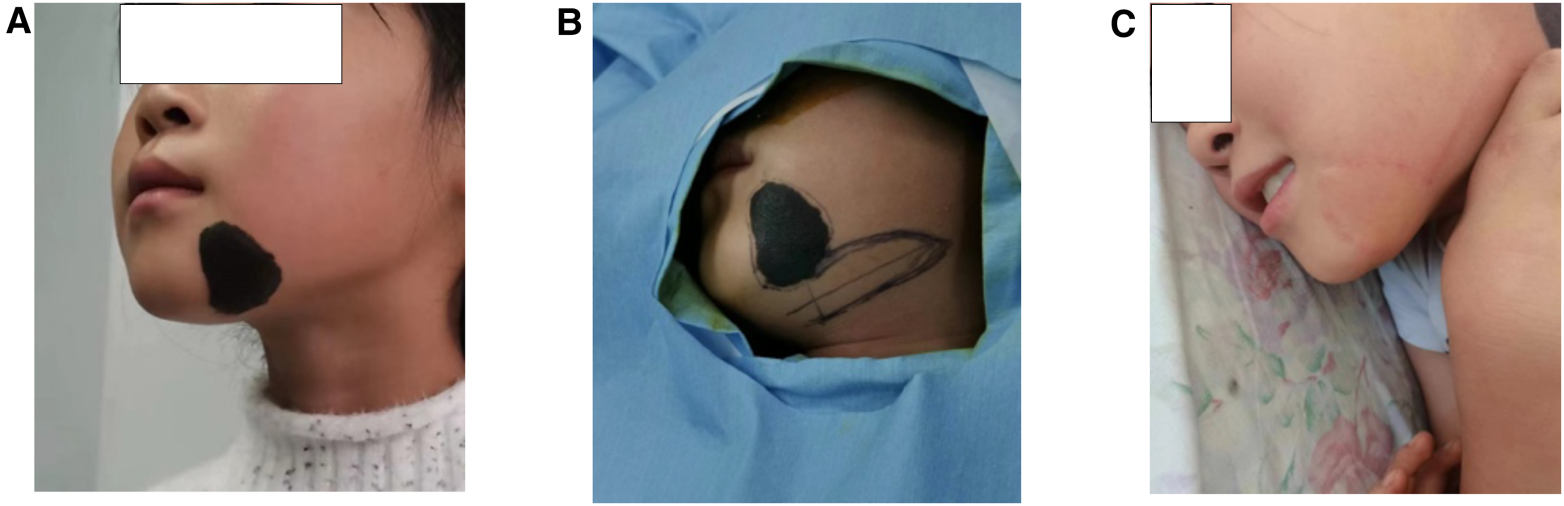
(**A**) Preoperative lesion skin. (**B**) Design of excision and flap area. (**C**) 10 months after the surgery.

An 9-year-old girl presented with melanin nevi (4.5*3.5 cm) on the lower left jaw region. She requested a single operation to achieve a good cosmetic result because of limited time. We designed a vertical transposition flap to cover her defect within the left aesthetic subunit of mandible. The patient recovered uneventfully and was satisfied with the final result (Figure 4).

## Discussion

**Figure 5:**
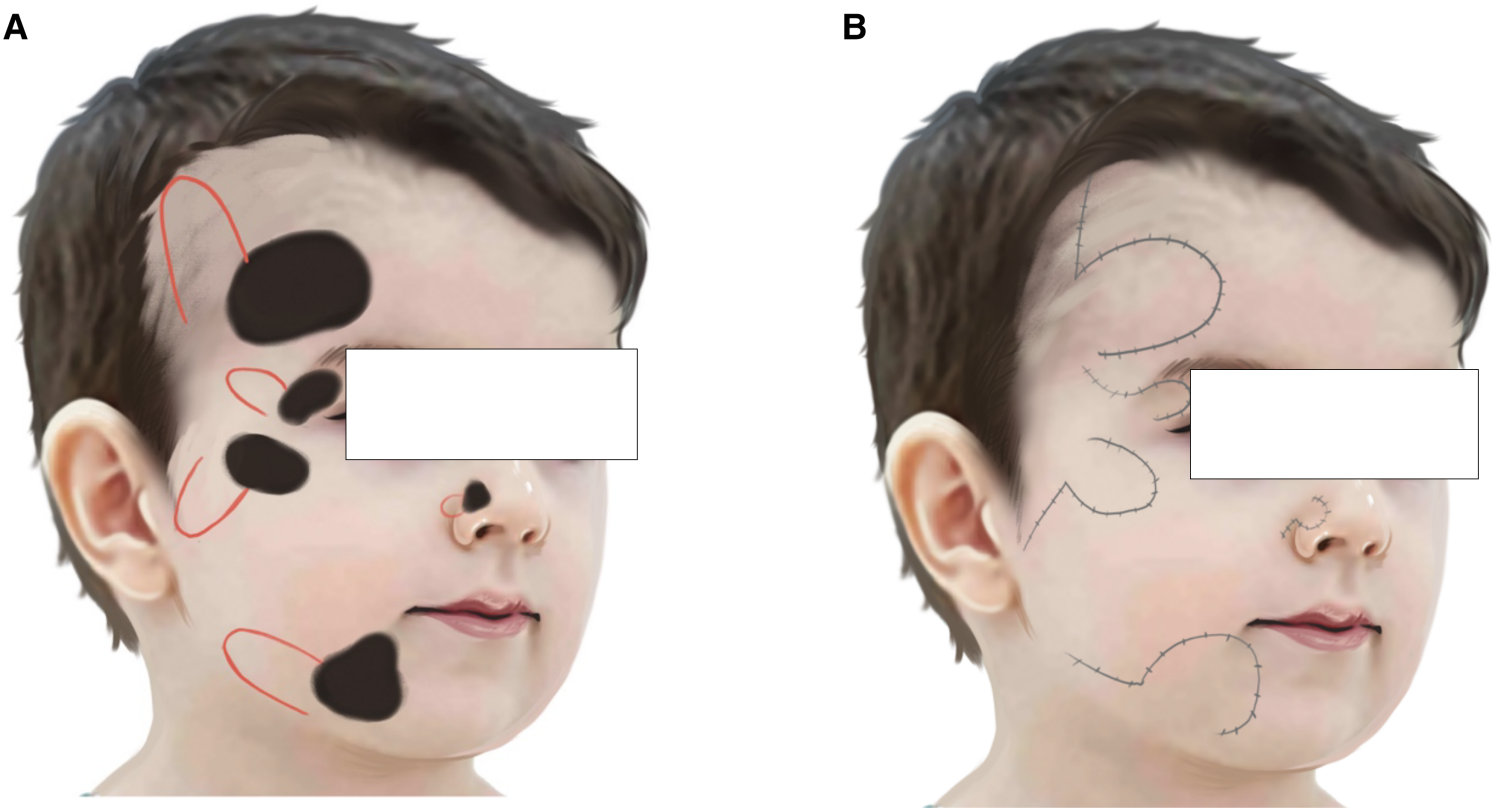
(**A**) Vertical flap design for different locations on face. (**B**) Secondary incisions should left in hairline, nasolabial fold, mandibular line or pre-auricular line.

Facial lesions or defects affect children's appearance, which would have long-term effects on the mental health and life quality of both children and parents (8, 10, 11). Special consideration should be given when reconstructing facial defects in pediatrics. Generally, the facial skin of children has more elasticity and is growing with the growth centers of the facial skeleton. It is difficult to conceal a surgical scar in the pediatrics as there are no distinct borders between aesthetic subunits. Thus, primary wound closure is less likely to produce an acceptable cosmetic result among children, especially for the large defects (12, 13). We use transposition flaps in pediatric patients with large facial defects rather than the most commonly used expansion flaps due to their higher complications. In addition, many parents request a single surgical including excision and construction for many reasons, such as limited time, cost, concerns about the damage to their child's central system development from multiple anesthesia. As powerful reconstructive tools, transposition flaps are frequently used in cutaneous reconstruction. During the closure of defects, transposition flaps borrow skin laxity from adjacent areas and redirect the vectors of tension. This allows the primary defect to be closed with a few or even no wound edge tensions (15). Transposition flaps have been modified over time and are now used in a variety of locations due to their versatility (16). Defects on forehead, eyelid, outer canthus, cheek and mandible allow surgeons to position the flap in hairline, nasolabial fold, mandibular line or neck. So it's easier for surgeons to conceal the flap incision scars and reduce the occurrence of pulling of the facial features (Figure 5). We found that flaps located in eyelid and nose can cover smaller defects and are more likely to show distraction of eyelid and lips.

Transposition flaps were frequently to be rotated between 45° and 90°. However, there was a tension overlap between primary and secondary defects if the angle was too small, resulting in limited extent of flap and heavy scar (17, 18). When the transposition angle was larger than 90°, the effective length of the flap would decrease significantly and a larger flap was thus needed (15, 16). In our center, we increase the transposition angle to 90°. It decreased the tension between flap and defect while having a larger flap. In addition, we found that the donor site and the defect can be closed more easily. Our results showed that most of children achieved a satisfied cosmetic and functional outcome. However, it should be noted that the increased angle will lead to an increased flap length and a higher incidence of flap necrosis (17). In our case series, 6 patients suffered from partial or full-thickness skin necrosis. Even though they recovered well in the end after careful management, skin necrosis is an important complication that should be pay attention to.

Important points of operation in our center were as follows: (1) Flaps selection: The defects should be repaired in the same facial subunit with local flaps. Cross-facial aesthetic unit flaps like expanded flaps were utilized for larger defects only when local flaps are not sufficient (18). Efforts should be made to feel for areas with a greater laxity and examine the effects of different tension vectors on adjacent skins. (2) Flap position selection: The long axis of skin flap should be placed along relaxed skin tension line (RSTL) so that the incisions can be less conspicuous. The incision should be left in the concealed part like hairline, nasolabial fold, mandibular line, pre-auricular line for greater scar camouflage. (3) Flap design: To ensure the survival of distal part of flap, the length-width ratio of the flap should be less than 3:1. The length of flap is the sum of long axis and 1/2 of the short axis. (4) Skin flap thickness: While thinner flaps usually allow better cosmetic facial landmarks, a certain thickness of fat layer should be preserved to avoid the damage of subdermal vascular network. (5) Dog-ear deformities: Significant dog-ear deformities should be corrected appropriately. However, the minor deformities could be preserved to avoid insufficient blood supply. (6) Postoperative caring: A surgical drain or needle aspiration could be applied for the drainage of accumulated blood or fluid under the flap (13, 19).

In conclusion, vertical transposition flap is effective in repairing large facial soft tissue defects in children, showing acceptable functional and aesthetic outcomes. However, this technique is far from perfect. Careful selection of appropriate patients and flap design might be needed.

## Data Availability

The original contributions presented in the study are included in the article/Supplementary Material, further inquiries can be directed to the corresponding author/s.
